# The World Coral Conservatory (WCC): A Noah's ark for corals to support survival of reef ecosystems

**DOI:** 10.1371/journal.pbio.3000823

**Published:** 2020-09-14

**Authors:** Didier Zoccola, Nadia Ounais, Dominique Barthelemy, Robert Calcagno, Françoise Gaill, Stephane Henard, Ove Hoegh-Guldberg, Max Janse, Jean Jaubert, Hollie Putnam, Bernard Salvat, Christian R. Voolstra, Denis Allemand

**Affiliations:** 1 Centre Scientifique de Monaco, Monaco; 2 Institut océanographique, Fondation Albert Ier de Monaco, Monaco; 3 Océanopolis, Brest, France; 4 CNRS INEE, Paris, France; 5 NAUSICAA, Centre National de la Mer, Boulogne sur mer, France; 6 Coral Reef Ecosystems Laboratory, School of Biological Sciences, The University of Queensland, St. Lucia, Australia; 7 Royal Burgers’ Zoo, Arnhem, The Netherlands; 8 Bio-Eco Sciences & Technologies, Nice, France; 9 Department of Biological Sciences, University of Rhode Island, Kingston, Rhode Island, United States of America; 10 Université de Perpignan, CRIOBE Moorea, USR 3278 EPHE-CNRS-UPVD, Moorea, French Polynesia; 11 Department of Biology, University of Konstanz, Konstanz, Germany

## Abstract

Global change causes widespread decline of coral reefs. In order to counter the anticipated disappearance of coral reefs by the end of this century, many initiatives are emerging, including creation of marine protected areas (MPAs), reef restoration projects, and assisted evolution initiatives. Such efforts, although critically important, are locally constrained. We propose to build a “Noah's Ark” biological repository for corals that taps into the network of the world’s public aquaria and coral reef scientists. Public aquaria will serve not only as a reservoir for the purpose of conservation, restoration, and research of reef-building corals but also as a laboratory for the implementation of operations for the selection of stress-resilient and resistant genotypes. The proposed project will provide a global dimension to coral reef education and protection as a result of the involvement of a network of public and private aquaria.

## Abstract 2

Global change is causing a widespread decline in coral reefs. This Community Page article proposes to build the World Coral Conservatory, a “Noah's Ark” biological repository that taps into the network of the world’s public aquaria and coral reef scientists, in order to preserve the fast-disappearing biodiversity of coral reefs.

## Introduction

Reef-building corals play a pivotal role in marine ecosystems and constitute the foundation of coral reef ecosystems that represent the world’s most important biogenic structures. Coral reefs provide habitat and trophic support for one-third of all marine organisms, and their biodiversity rivals that of tropical rainforests [1]. In addition to their ecological importance, coral reefs are economically essential for many human societies, with more than 600 million people around the world depending directly on coral reefs for their survival [2–4]. The total annual value of global coral reefs is estimated to be more than US$30 billion/year [5, 6]. However, because of a combination of local and global stressors (pollution, overexploitation of resources, diseases, and climate change), marine ecosystems outpace terrestrial habitats in biodiversity erosion [7]. Coral reefs, the frontrunners of this trend, are declining at an unprecedented rate, making them the most endangered biological systems [8]. De’ath and colleagues have shown that the growth rate of reefs decreased by 15% over a 20-year period [9], and Hughes and colleagues [10] estimated that we lost about 30% of corals from the Great Barrier Reef because of a single coral bleaching (2015–2018) event. Even more alarming, the most recent Intergovernmental Panel on Climate Change (IPCC) special report “Global warming of 1.5°C”, released in late 2018, anticipates a likely loss of 90% of reef-building corals by 2100 under a warming scenario of +1.5°C and a virtually complete loss of corals (>99%) under a warming scenario of +2°C [11]. Although certain coral species may have physiological and genomic attributes that make them more resilient/resistant to heat stress [12], a substantial loss of corals and the reefs they build is inescapable. Consequently, we need to establish interventions (for example, [13, 14]) in order to avoid the unmanageable and manage the unavoidable.

Both in situ and ex situ (on-land) actions can be taken to protect and/or restore coral reefs, and both approaches have their advantages and drawbacks as outlined in the following. The vast majority of conservation actions are in situ protection, either through the establishment of marine protected areas (MPAs) or through restoration of degraded coral reefs. The importance and benefits of MPAs are well documented, serving primarily to decrease local stress to increase the resilience of reefs [15–17]. Unfortunately, MPAs currently encompass less than 10% of the surface area of coral reefs [18], and studies suggest that MPAs are limited in their protective scope [19]. Further, MPAs largely protect coral reefs from local threats, but not from global stress. Therefore, they may prove ineffective in the face of climate change. As outlined above, from 2015–2018, the northern part of the Great Barrier Reef, despite being protected and remote from direct anthropogenic impacts, had lost an estimated 30% of its surface of living corals because of coral bleaching triggered by ocean warming [10]. Coral reef restoration has also become an active area of coral reef management [20]. Though local actions are currently limited to small areas (on the order of square kilometers), technological developments could facilitate restoration of larger areas in the future. For instance, the use of more resilient/resistant coral colonies as source material for reef restoration has been suggested, yet it is unclear how thermal tolerance may shift under large-scale warming for extended periods and under the increase in the severity and frequency of marine heatwaves [21, 22]. Consequently, efforts to rebuild reefs through restoration efforts should incorporate knowledge on current and projected resilience/resistance of the corals used for that purpose (see also below).

To address the problem that not all reefs can be saved and to focus efforts on reefs that are most likely to survive in the future, Hoegh-Guldberg and colleagues conducted a global scale analysis to identify resilient regions for which long-term coral reef conservation may be an attainable goal, even under ongoing ocean warming (“50 Reefs Initiative,” [23]). The identified regions are putatively less susceptible to impacts from thermal stress and coral bleaching [23], such as the Persian Gulf [24, 25] or the Red Sea [26–29]. Additionally, different types of reef “oases” were identified by Guest and colleagues [30] in a large-scale comparison of multidecadal time series data, further highlighting reefs of interest with respect to resilience, conservation, and restoration. Although not universal [31, 32], the mesophotic zone between 30 and 150 m [33] is another possible refuge area. Indeed, as shown by Kramer and colleagues [34], its upper part can serve as a refuge for corals and become a source of larvae that can facilitate the recovery of shallow degraded areas, although it is not clear in how far shallow and mesophotic reefs system are genetically connected [32]. Notably, such sites are for the most part identified based on remote-based approaches and observational surveys, disregarding the substantial variation that exists at the level of individual coral colonies/genotypes [35–37].

An alternative for in situ conservation management would be to use ocean warming resilient/resistant specimens for reef restoration purposes. However, natural populations of resilient/resistant corals, such as those from the Persian Gulf, are locally adapted and likely do not prevail in other environments [38, 66]. Given the urgent need to mitigate coral reef declines and the limitations of classic conservation and restoration measures, Van Oppen and colleagues [39] proposed using assisted evolution methods, inspired by more than 10,000 years of selection of resistant strains and environmental hardening approaches followed in agriculture and aquaculture. Briefly, the authors propose to promote resilience/resistance of coral colonies by (1) inducing laboratory stress and selecting the coral colonies that survive, (2) actively modifying the coral-associated microbiota, (3) applying environmental stress hardening to generate more resistant phenotypes, and (4) by genetically enhancing coral host-associated microalgae (Symbiodiniaceae, [40]) by means of mutation and selection using artificial evolution (see also [41–43]). Subsequently, methods for active modification of the coral genome through approaches such as CRISPR and synthetic biology [14] were suggested. These methods require the consideration of both logistical and ethical challenges [44, 45] before field implementation should be attempted.

On-land (ex situ) solutions aim to preserve corals outside their natural environment, with the benefit that such maintained corals are not subject to marine heatwaves or other natural events (for example, hurricanes) that might destroy them. Rather, ex situ solutions provide a more controlled environment where corals are not subject to arbitrary environmental change. At the same time, we acknowledge the diversity of opinion around this topic, and the concern that aquaria conditions ultimately “select” for domestic rather than wild genetic profiles. In other words, corals that are well adapted to “benign” aquaria conditions will do better, which may make it challenging to maintain coral genotypes under aquaria settings that respond well to environmental extremes. The ultimate utility of aquaria as a coral conservatory (and seedbank) will have to be demonstrated, and the best way to do this is by incorporating “the science learned along the way”. Similar to what has been done with vertebrates [46] or crops/plant species [47], initial approaches in corals have entailed the construction of a “vault” to store germinal cells from different coral species to preserve the genetic information of a species and/or particular genotypes. For instance, Hagedorn and colleagues [48–50] successfully cryopreserved coral spermatozoa cells and dissociated embryonic cells. Another alternative is to cryopreserve coral larvae or so-called tissue balls [51, 52]. However, cryopreservation was shown to reduce fertilization success [53] and the techniques are not available off the shelf, with improvement required to be applicable in a standardized and broad manner. Also, further research is needed to assess and understand putative long-term effects and success.

Ex situ culturing builds on the conservation work already carried out by zoos and aquaria for endangered populations of vertebrates and invertebrates [54, 55]. This approach employs a network of aquaria with the benefits of (1) conserving coral species, (2) generating coral material without depleting wild stocks for research into areas such as assisted evolution, and (3) providing a multispecies test bed for the performance and interaction of manipulated corals prior to ex situ culturing and field testing. Given that coral culturing is occurring in numerous research institutes and public aquaria, along with methods of propagating corals through sexual and asexual reproduction also being developed [56], a network of aquaria provides a critical global resource for threatened corals. Of note, in light of the diversity of aquaria water supplies, daily settings, and vertebrate and invertebrate species diversity, such asexually propagated coral fragments will be exposed to a variety of environmental conditions, which should reduce concern for limited plasticity, thus avoiding domestication of corals to an artificial environment.

## Aim(s) of the World Coral Conservatory (WCC) project

**Fig 1:**
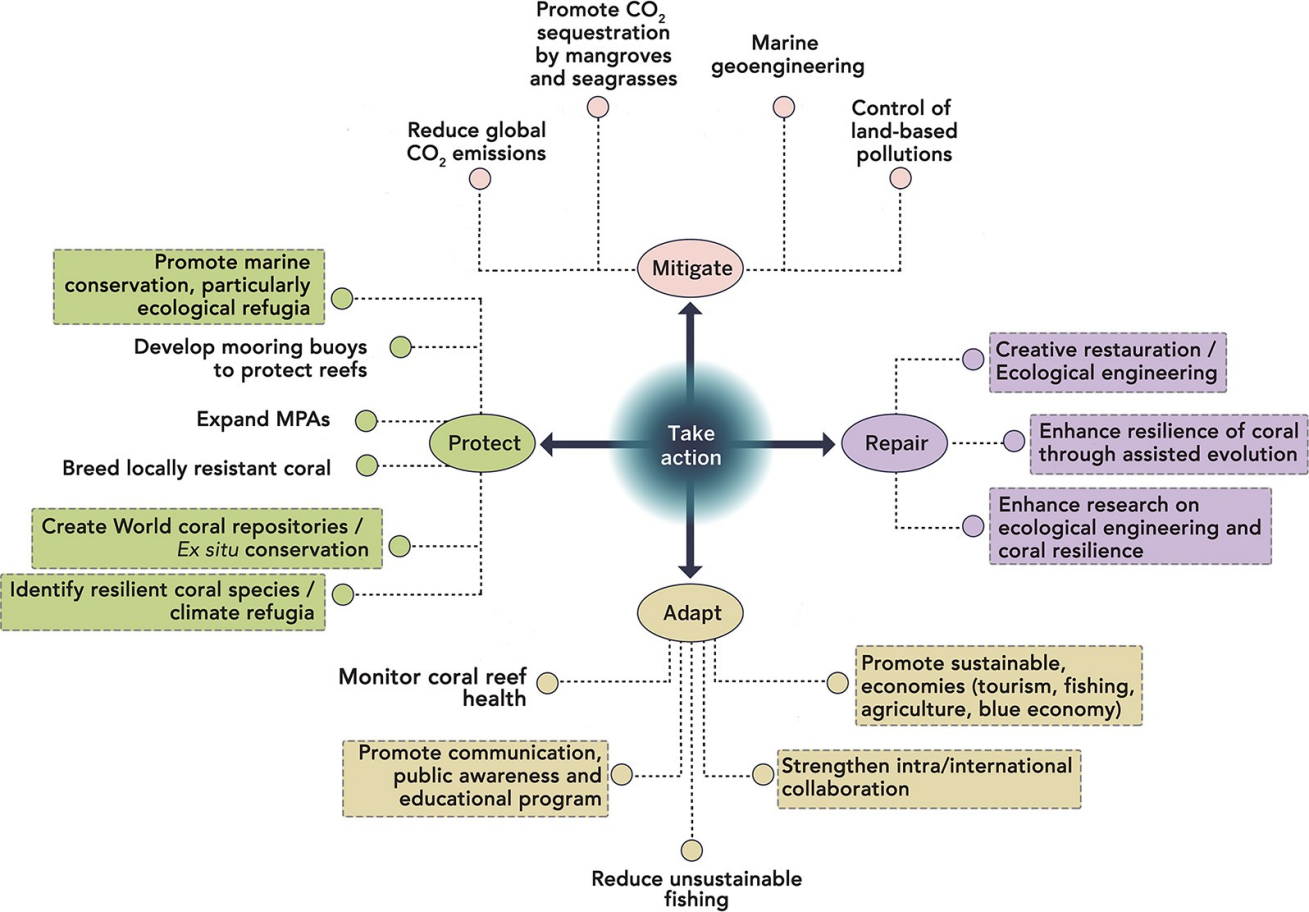
Solutions for coral reefs against climate change impact supported through the WCC project. The diagram is based on the 4 clusters of actions proposed by Allemand and Osborn [57] for coral reef ecosystems. Specific solutions supported the WCC are highlighted by colored squares. WCC, World Coral Conservatory.

**Fig 2:**
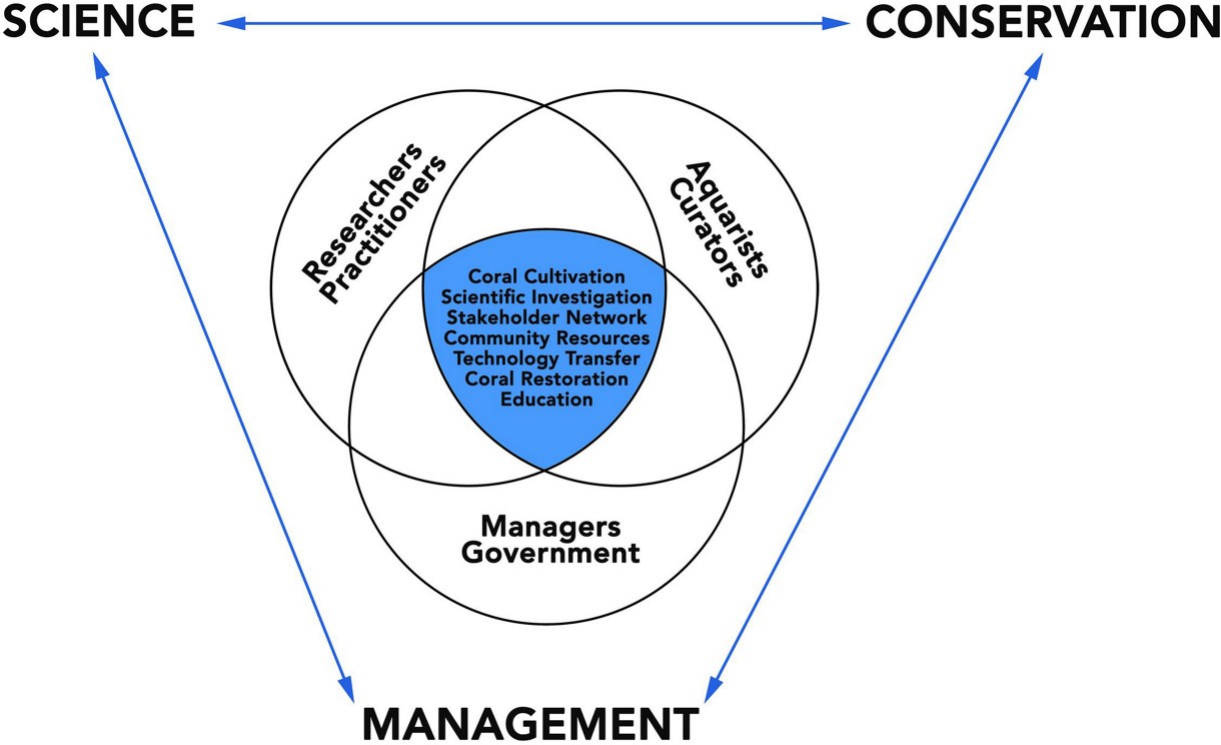
The WCC is based on a consortium of researchers, aquarium curators, and field managers facilitating the transfer of information between the 3 main components. MPA, marine protected area; WCC, World Coral Conservatory.

Public aquaria around the world now maintain and present live coral and ensure continuous replication/persistence through so-called cuttings. Emerging from discussions at the “International Coral Reef Symposium 2016” (ICRS2016) in Honolulu (Hawai’i, USA), we propose here the creation of the WCC. This endeavor would take advantage of combining 3 of the 4 safeguarding actions proposed by Allemand and Osborn [57], supporting protection, adaptation, and reparation as solutions for coral reefs against climate change impact (Fig 1). The WCC takes advantage of the existing global network, infrastructure, and facilities of public aquaria as a means to protect the biodiversity of coral reefs via the maintenance, propagation, and study of aquaria-reared reef-building corals. Our solution is based on 3 components: Science, Conservation, and Reef Management (Fig 2).

The aims of the WCC are as follows:

- To create a repository of living coral colonies that represent the majority of extant species;
- To preserve coral genetic and species diversity from a variety of habitats and by this a mechanism to contribute to restore coral reef diversity through reintroduction and reef restoration approaches [58];
- To contribute to protecting coral reef biodiversity by developing solution-based approaches that combine reef science, conservation, and management;
- To provide researchers from all over the world with referenced and trackable biological material, which also reduces the burden of collection of biological material from coral reef ecosystems and supports standardization via research on shared resources (same coral genotypes);
- To use the available biological resources for "assisted evolution approaches" to increase stress tolerance and resilience/resistance; and
- To provide comprehensive information on corals and coral reefs to the general public and stakeholders in order to educate and enable people to participate in the collective effort of coral reef conservation. The IPCC *Special Report on the Ocean and Cryosphere in a Changing Climate*, approved in Monaco in September 2019, highlights education as an essential component as a solution for safeguarding ecosystems.

The urgent need to preserve the unique biodiversity and natural heritage of corals and to minimize harvesting corals from the field for scientific or commercial purposes makes approaches such as the WCC a priority. Notably, we will prioritize coral species based on geographical distribution and population size. For the biological repository, which we term the “coral bank”, state-of-the-art genetic techniques will allow the precise identification of species and genotypes in order to trace colonies from their respective origin and to monitor distribution and spread of daughter colonies (i.e., ramets) for research and propagation. Preservation of genetic diversity within species and populations in the WCC via genotype identification, archiving, and tracing is a prerequisite for successful conservation measures and currently not widely implemented in coral restoration practices.

## Methodology and roadmap

The first meeting of the steering committee of the WCC was held in 2019 during the Monaco Ocean Week, and we defined a plan to sample, cultivate, investigate, develop, communicate, restore, and transfer (Fig 2).

**Fig 3:**
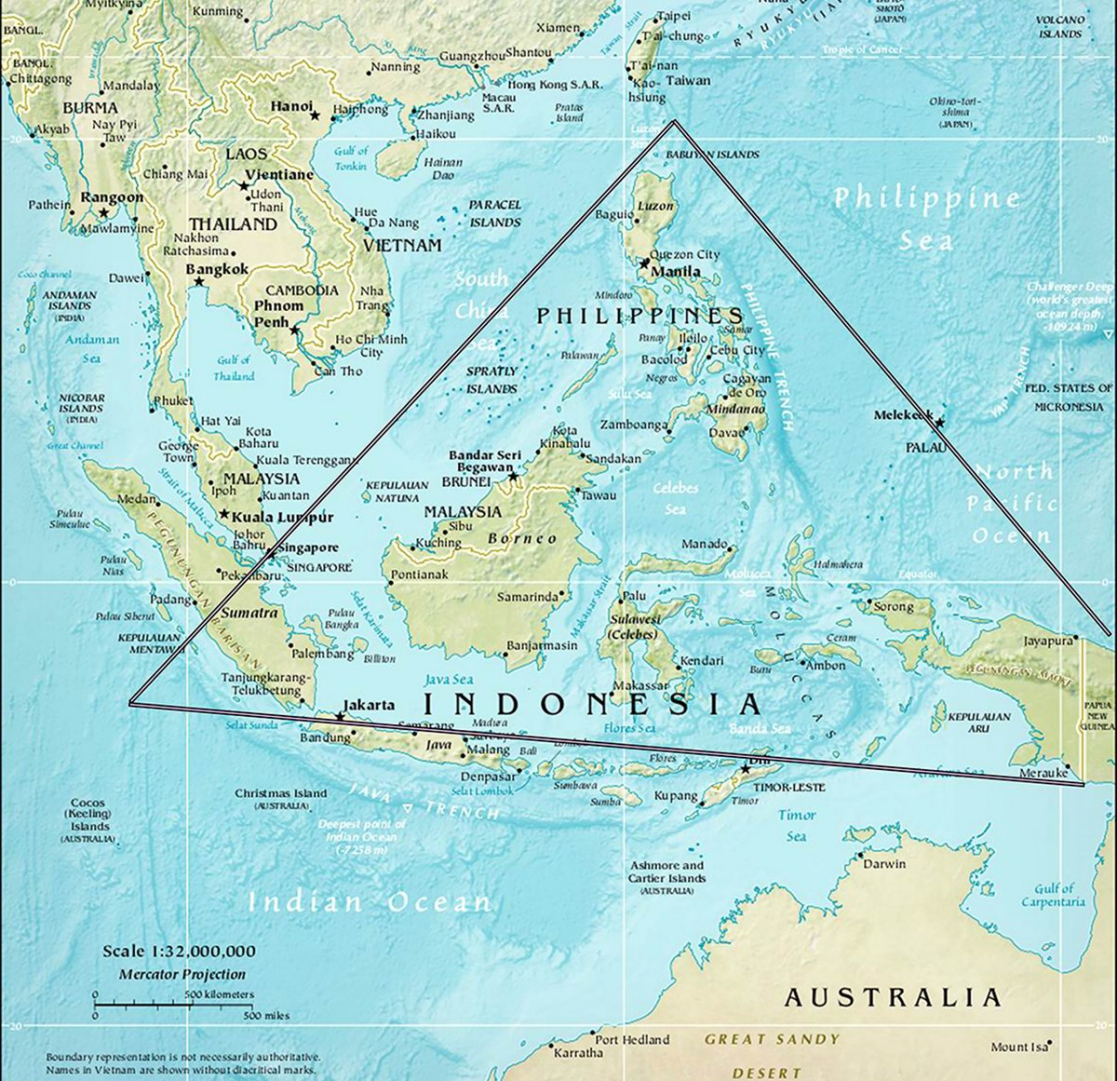
The coral triangle denotes the geographical area in the Pacific between Indonesia, the Philippines, and Papua New Guinea and is home to the largest coral species biodiversity in the world; i.e., about 40% of the world coral species are represented in this area. Map from Worldofmaps.net.

The number of coral species currently kept in aquariums is on the order of 250, which represents only a small fraction, around 15%, of the estimated 1,600 coral species globally [59], highlighting the challenge of deciding which species to choose. Should the choice be made between putative climate-resilient/resistant species or between iconic species best representing coral reefs? For instance, corals from the Arabian Seas presumably have increased thermotolerance and are more resistant to bleaching. At the same time, the number of species in the Persian Gulf is limited (about 70 in total). Hence, by choosing a relatively narrow set of expected “winners”, this approach risks eroding global biodiversity [60]. It is therefore essential to take a balanced approach to species selection, which includes first, broad genetic diversity, and second, corals with a variety of specific traits (for example, thermal tolerance, high fecundity, disease tolerance) and from a variety of habitats, shown to contribute to coral performance [61]. The overarching goal is to capture a breadth of genetic diversity to reduce the potential for bottlenecks or tradeoffs that may come with a focus on a few tolerant species or genotypes. To support the initial goal of biodiversity, species will need to be sampled broadly across many locations. Based on our experience from the *Tara* Pacific Expedition [62] and in order to ensure a breadth of genetic diversity, colonies from each species will be collected in 5 different locations. One of the first regions of interest is therefore the most biodiverse geographic area, the coral triangle (Fig 3). This region contains about 600 species of scleractinian corals out of the 1,600 global species. Furthermore, many countries in this region have the infrastructure and skills to help support coral sampling and global coral dispatching efforts. Indeed, other locations will be chosen, such as the Indian Ocean, the Red Sea, and the Great Barrier Reef. Therefore, in order to maximize the areas where we will be able to collect corals, we have decided to join forces and team up with initiatives that are close to our objectives. These global initiatives include the Great Barrier Reef Legacy (https://greatbarrierreeflegacy.org/), the Association of Zoos and Aquariums, the National Oceanic and Atmospheric Administration, and the Mote Marine Laboratory, which are participating in the Florida Reef Tract project (https://www.aza.org/the-florida-reef-tract); further, the Smithsonian Conservation Biology Institute in Hawai’i.

**Fig 4:**
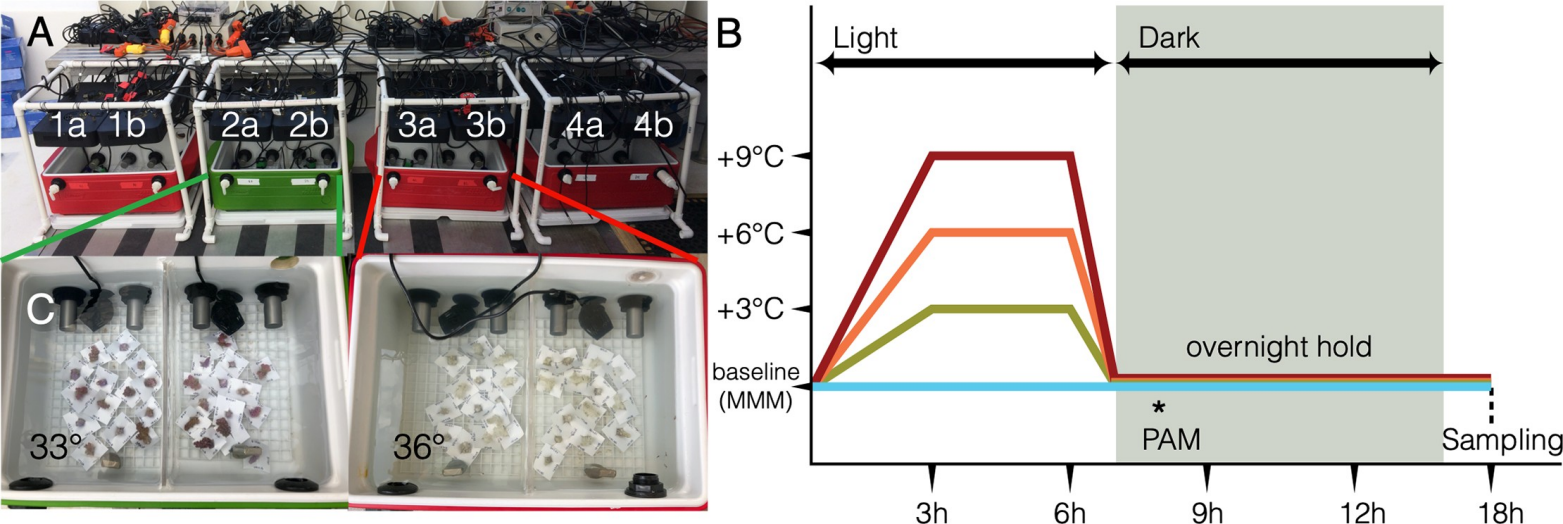
Overview of the CBASS. (A) Picture from Christian R. Voolstra showing CBASS featuring 4 light- and temperature-independent, flow-through tanks with 2 replicate units (1a/b: baseline temperature based on MMM of tested site, 2a/b: MMM +3°C, 3a/b: MMM +6°C, 4a/b: MMM +9°C). (B) 18-hour–long programmed temperature profile for assessment of coral thermal tolerance thresholds. Asterisk = measurement of photosynthetic efficiency as proxy for coral health (see [70]). CBASS, Coral Bleaching Automated Stress System; MMM, maximum monthly mean; PAM, Pulse-Amplitude Modulation.

Our second goal is to perform a targeted collection of corals with specific traits. Endemic species should be part of the priority list, given their unique cultural value and potentially interesting genetics and physiology [41, 63, 64]. For example, the Caribbean area, which is under rapid decline, has only 62 described species, but 43 of them are endemic to the region, while endemic Hawaiian species show unique gene family expansion and gene duplication features [65] that will provide insight in comparative studies. Another strategy is to collect tolerant/resistant/resilient genotypes to specific environmental parameters such as thermal stress. The challenge remains how to identify such colonies. While long-term heat stress experiments are useful tools to identify resilient/resistant genotypes and have a track record in the literature [66], they require extensive and expensive aquarium systems and are not practical in remote field locations and settings. Further, the approach takes weeks to months for an assessment of just a single set of individuals from a single population. Recent experiments utilizing short-term acute thermal exposures in remote field settings show a promising ability to reveal fine-scale differences in thermal tolerance across small spatial scales [66–68]. Based on these experiences, Voolstra and colleagues [69] have demonstrated the suitability of standardized short-term acute heat stress assays to resolve intraspecific differences in coral thermotolerance in 2 populations from contrasting thermal environments separated by <500 meters. The Coral Bleaching Automated Stress System (CBASS), a portable, inexpensive, standardized experimental system developed by Barshis, will be used to select stress-resilient colonies for aquaria maintenance ([69] and Fig 4). It uses multiple temperatures to screen for coral colonies with resilient/resistant phenotype characteristics within 1.5 days and allows alignment of determined phenotypes with the underlying genetic/genomic makeup via subsequent molecular analysis. The screening can be extended to incorporate other traits of interest, such as tolerance towards high nutrient levels or other anthropogenic impacts. An increased ability to determine which corals are more resilient/resistant to stressors and their underlying holobiont features (genes, gene variants, Symbiodiniaceae types, associated bacteria) will help to focus efforts on specific genotypes and sites and inform action on conservation and restoration efforts.

Because it is paramount that samples are uniquely identified and tracked in their respective native environment, we propose to use transponders for this purpose, as is done, for example, for penguins [70]. The barcode assigned to each colony will be added to the identity card of the colony, which will identify the species by in situ photos, a geographical location of the sample, a genetic barcode, photos of the skeleton, etc. Each colony sent to aquaria or research laboratories will thus be followed and can be related back to its origin.

In addition to the aquarium of Monaco, which was the first in the world to present a life-size living coral reef, public aquariums are now successfully maintaining and cultivating live corals, for example, Océanopolis (France), Nausicaa (France), and Burgers' Zoo (The Netherlands). In total, we anticipate about 50 aquariums to comprise the network of the WCC.

For all coral species that are collected under the WCC, we will provide an assessment of genetic diversity based on collected coral colonies. Further, a reference transcriptome (and genomes where possible) will be produced for each species to address questions regarding the following:

- Biodiversity: with more than 13,000 species, the sequencing of nearly 10% of Cnidarians will provide us with a good overview of the species that comprise the phylum Cnidaria;
- Evolution: availability of 1,000+ transcriptomes allows us to address fundamental questions of coral evolutionary biology, such as the formation of new species, adaptive radiation, calcification, symbiosis, and the genetics of adaptability in light of climate change;
- Biomedical and cosmetic applications: corals offer very interesting aspects concerning aging and regeneration, for which the sequencing of 1,000 transcriptomes will improve our knowledge regarding candidate genes contributing to longevity and the extraordinary resistance to UV exposure. Further, corals can become ecological sentinels, and tests using coral cultures can become an industrial standard of ecotoxicity for cosmetics and other xenobiotics [71];
- Biomonitoring: the recent use of environmental Deoxyribonucleic Acid (eDNA) is a major breakthrough [72] because organisms leave DNA traces/fingerprints in their environment, especially seawater. This DNA can be extracted from seawater and the composition of the species present at the site where the water was collected can be determined [73, 74]. However, a data library containing barcodes for each species is needed. The 1,000 transcriptomes will allow for doing this.

The sequencing efforts will result in a massive amount of valuable data for the scientific community and will be made publicly available along with the tracking and metadata.

The WCC is also interested in applying assisted evolution approaches [39] using the available biological material because the ability to develop and maintain improved coral stocks will support the restoration of coral reefs, with hopefully improved resilience and survival. The coral stocks obtained would also provide another source of animals for the home aquarist industry, which would in turn reduce the pressure of collection on natural reef sites.

We think that it is also important to have a network of non-governmental organizations (NGOs) working on reef restoration, such as the “50 Reefs initiative” (Ocean Agency, Global Change Institute at The University of Queensland, and the Wildlife Conservation Society), which aims to identify, preserve, and restore 50 coral reefs around the world as priority reefs for conservation [22]. The WCC, by communicating with this new consortium, could provide local corals when regions become devastated by repeated bleaching events.

The WCC, via its public aquarium network, can play a prominent role in educating various audiences about the state of scientific research, the specific biology of corals, the health state of reefs around the world, their contribution to the world's biodiversity, and the services they provide to humans.

Finally, capacity building is an important aspect to this project; transfer of knowledge and techniques on coral culture (microfragmentation, for example [75]) is necessary to increase the number of public aquaria globally that are able to cultivate corals. This is an important way to contribute to coral restoration because the provisioning of corals is significantly facilitated if the supply site is near the restoration site given that the transport of corals is a delicate and expensive operation. In addition, the corals can act as a novel resource for other public aquaria to obtain corals while avoiding collection of new coral colonies from the wild.

The WCC contains many areas of interest to industry and, in particular, holds promise for the development of biomedical and cosmetic applications. The involvement of industrial actors should provide financial resources to support research and development, which will provide a link for industry to follow their interests but, at the same time, share part of the responsibility to maintain coral reefs as an ecological and economical resource for future generations.

## Conclusion

We here lay out the overall vision and mission of the WCC (World Coral Conservatory), an internationally open community of research laboratories, science centers, public aquaria, stakeholders, and national administrations that builds on the idea to use aquaria as a “Noah’s Ark” to preserve corals and to establish a global network and platform for sharing of biological material, and for knowledge generation and exchange to enhance and support conservation and restoration of coral reef ecosystems. This network is based on the principles of sharing of data, organization of conferences, symposia, seminars, congresses, temporary exhibitions, and internet forums and welcomes participation and contribution of industry, schools, associations, divers, and citizens. We set out a call to the community to facilitate the pooling of resources and federate stakeholders around common projects for the good of coral reefs, as proposed by the Coral Bleaching Research Coordination Network (https://u.osu.edu/grottoli.1/coral-bleaching-rcn/). These concerted efforts could then provide decision-makers (governments, communities, local and institutional elected officials), reef-area managers, and users with relevant tools in terms of information, methodologies, and decision support. The global network of public aquaria to house valuable coral reef resources provides an essential link between conservation, research, and education efforts.

## Abbreviations

CBASS: Coral Bleaching Automated Stress System
eDNA: environmental Deoxyribonucleic Acid
IPCC: Intergovernmental Panel on Climate Change
MPA: marine protected area
NGO: nongovernmental organization
WCC: Word Coral Conservatory
